# A Peptide to Reduce Pulmonary Edema in a Rat Model of Lung Transplantation

**DOI:** 10.1371/journal.pone.0142115

**Published:** 2015-11-04

**Authors:** Klaudia Schossleitner, Andreas Habertheuer, Richard Finsterwalder, Heinz P. Friedl, Sabine Rauscher, Marion Gröger, Alfred Kocher, Christine Wagner, Stephan N. Wagner, Gottfried Fischer, Marcus J. Schultz, Dominik Wiedemann, Peter Petzelbauer

**Affiliations:** 1 Department of Dermatology, Skin and Endothelium Research Division (SERD) Medical University of Vienna, Vienna, Austria; 2 Department of Cardiac Surgery Medical University of Vienna, Vienna, Austria; 3 Core Facility Imaging Medical University of Vienna, Vienna, Austria; 4 Department of Dermatology, Division of Immunology, Allergy and Infectious Diseases Medical University of Vienna, Vienna, Austria; 5 Department of Blood Group Serology and Transfusion Medicine, Medical University of Vienna, Vienna, Austria; 6 Department of Intensive Care & Laboratory of Experimental Intensive Care and Anesthesiology (L.E.I.C.A), Academic Medical Center, University of Amsterdam, Amsterdam, The Netherlands; University of Colorado Denver, UNITED STATES

## Abstract

**Background:**

Despite significant advances in organ preservation, surgical techniques and perioperative care, primary graft dysfunction is a serious medical problem in transplantation medicine in general and a specific problem in patients undergoing lung transplantation. As a result, patients develop lung edema, causing reduced tissue oxygenation capacity, reduced lung compliance and increased requirements for mechanical ventilatory support. Yet, there is no effective strategy available to protect the grafted organ from stress reactions induced by ischemia/reperfusion and by the surgical procedure itself.

**Methods:**

We assessed the effect of a cingulin-derived peptide, XIB13 or a random peptide in an established rat model of allogeneic lung transplantation. Donor lungs and recipients received therapeutic peptide at the time of transplantation and outcome was analyzed 100min and 28 days post grafting.

**Results:**

XIB13 improved blood oxygenation and reduced vascular leak 100min post grafting. Even after 28 days, lung edema was significantly reduced by XIB13 and lungs had reduced fibrotic or necrotic zones. Moreover, the induction of an allogeneic T cell response was delayed indicating a reduced antigen exchange between the donor and the host.

**Conclusions:**

In summary, we provide a new tool to strengthen endothelial barrier function thereby improving outcomes in lung transplantation.

## Introduction

Lung transplantation (LTX) has become an effective treatment option for end-stage lung diseases, with 1-year survival rates around 80%. Despite significant improvements, up to 50% of patients develop primary graft dysfunction (PGD), resulting in prolonged length of mechanical ventilation, prolonged intensive care unit and hospital stay, increased cost, and increased morbidity and mortality [1]. PGD occurs during the first 72 h post LTX and is characterized by progressive pulmonary edema with diffuse alveolar damage that manifests clinically as hypoxemia.

Fluid exchange between the vascular system and tissues is tightly regulated to maintain the normal homeostasis in healthy individuals. During surgery, mechanical ventilation, systemic inflammation and specifically during LTX, the system gets poised towards a loss of vascular integrity resulting in vascular leak [2, 3]. This causes perivascular tissue edema impeding tissue oxygenation and nutrition. Specifically in the lung, edema between pulmonary capillaries and alveoli reduces gas exchange not only damaging the lung itself, but also causing systemic hypoxia and inflammation [4]. This may lead to delayed organ function or even dysfunction requiring extended mechanical ventilation or extracorporeal membrane oxygenation [5, 6]. This results in higher perioperative mortality and morbidity such as infections, bleeding, or prolonged intensive care. It can also induce higher numbers of acute and chronic rejection episodes [7, 8].

At a cellular level, endothelial cells build the barrier between the vessel lumen and surrounding tissue. This function is accomplished by endothelial junctions building bridges between neighboring cells. Endothelial junctions built by VE-cadherin, so called adherens junctions, control permeability for cells and macromolecules [9]. Tight Junctions, built through 4-pass membrane proteins such as occludin and claudins, provide the paracellular permeability seal for small molecules and fluid [10, 11]. The function of tight junctions is under the control of cytoplasmic adaptor proteins. One of those proteins, called cingulin, is a prominent regulatory protein due to its actin-bundling activity [12] and due to its interactions with guanine nucleotide exchange factors (GEFs), such as GEF-H1 [13], p114RhoGEF [14] and MgcRacGAP. GEFs control the activation of RhoGTPases and in the context of tight junctions, are responsible for junction assembly and disassembly [15].

We have recently described a cingulin-derived synthetic peptide, XIB13, which prevents stress-induced membrane ruffling and actin bundling in vitro and reduces burn-induced tissue edema in vivo [16]. Employing our orthotopic lung transplantation model in rats [17], we show that XIB13-treated rats have decreased lung edema, improved blood oxygenation and preserved lung morphology as analyzed 100min and 28 days post-surgery.

## Materials and Methods

### Peptide synthesis

The XIB13 (GRRPLGGISGG) and control peptide (IGLGRPGGGRS) were produced synthetically by solid-phase peptide synthesis and purified with reverse-phase high-performance liquid chromatography using nucleosil 100-10C18 columns (UCB-Bioproducts). The endotoxin concentration was <0.06 EU/mg and microbial contamination was <1 colony-forming unit (c.f.u.)/100 mg.

### Rat model of lung transplantation (LTX)

All experiments performed in this study have been approved by the Institutional Committee for Animal Research and Care at the Medical University of Vienna (BMWF-66.009/0254-II/3b/2011). Animals were held under standard conditions with unlimited access to water and standard laboratory food. Male Fischer F344 rats weighing 250–300g were used as donors; male Wistar Kyoto WKY rats weighing 320–350g served as recipients. Animals were obtained from Charles River Laboratories (Sulzfeld, Germany) and were housed for at least 2 weeks under special pathogen free conditions prior to transplantation. All animals were orotracheally intubated, mechanically ventilated using a pressure control mode (Biegler Medizin Elektronik small animal ventilator) at 15mbar P_max_, 2–4mbar PEEP, 0.7 FIO_2_, 1.5l/min flow rate and anaesthetized using Ketamine (Ketasol®) 100mg/kg and Xylazine (Rompun®) 4mg/kg intraperitoneally. All recipient animals were additionally pretreated with 1mg/kg Flunixin (Finadyne®) s.c. and 0.2mg/kg Fentanyl (Fentamed®) s.c. to obtain best possible analgesia. Oxygenation rates were monitored using a pulsoxymeter.

The surgical technique of left orthotopic single lung transplantation has been described before [17]. In brief, donor lungs were harvested following systemic heparinization. XIB13 (GRRPLGGISGG) [16] or random peptide (IGLGRPGGGRS) (1mg in 0.5ml/lung) was administered via the pulmonary artery of the donor animals after flushing the lungs with cold preservation fluid of low potassium dextran glucose (Perfadex®) with 1μl/ml of sodium bicarbonate. Lungs were kept fully inflated on ice for 180min. The venous clamp was removed prior to the arterial clamp to avoid high pulmonary pressure and patency of the venous anastomosis was verified by the immediate retrograde pulmonary venous filling from the left atrium, as shown previously [17]. Recipient rats underwent left lateral thoracotomy in the 4^th^ intercostal space. After excision of the left native lung the donor lung was implanted by using an interrupted suture technique and a cuffing approach for the bronchus and the pulmonary vasculature, respectively. Reperfusion was initiated after an ischemia time of 180min. All animals received a single injection of 25mg methylprednisolon i.p. at the time of reperfusion and 4 times 1mg XIB13 or random peptide i.v. (20min before and at the time of reperfusion, and 20min and 60min thereafter). Five and 30 minutes postoperatively lungs were hyperinflated using a single-lumen intubation tube with 15mbar PEEP. Recipient animals were monitored for 100min (acute group) with the chest remaining open or 28 days (chronic group) following surgical chest closure. At respective time points lungs were flushed, explanted and cut into 4 transverse sections.

### Measurements in the acute model, 100min post LTX

#### Bronchus edema score

**Fig 1:**
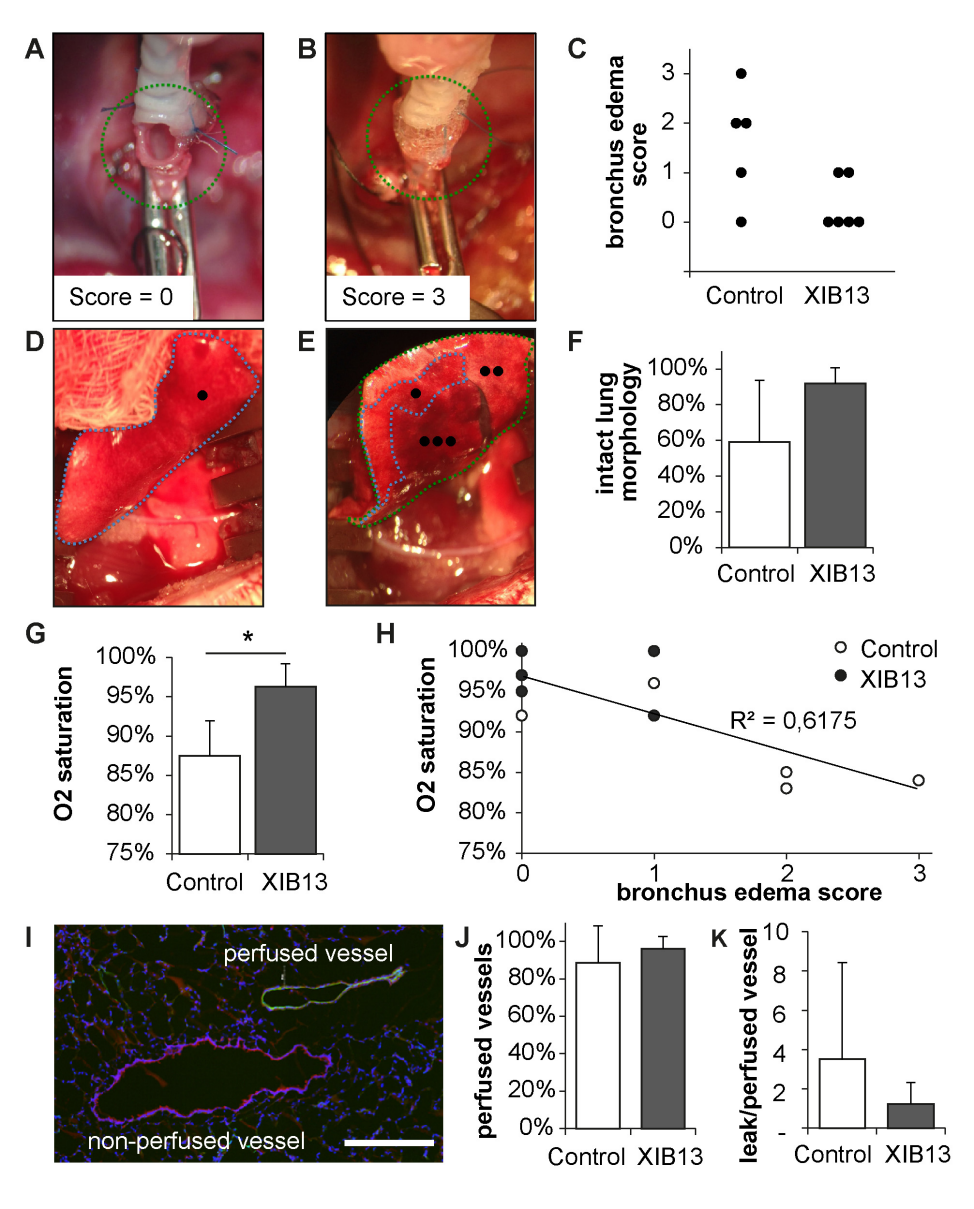
Improved oxygenation in XIB13-treated animals 100min after lung transplantation. Donor lungs were ex vivo perfused with XIB13 or scrambled peptide and put on ice until grafting. Thereby lungs were kept fully inflated with a tracheal clamp, equivalent to clinical lung transplantation. At the time of grafting, the tracheal clamp was released resulting in the spontaneous expulsion of fluid from the trachea in controls (exemplified in B) but not in XIB13-pretreated lungs (A). Results were summarized in (C, p = 0,082; n = 5/group). Lungs were explanted 100min after LTX. The perfused and ventilated areas were quantified macroscopically as exemplified in (D, XIB13-treated; E, control lung): areas marked by “•”were homogenously perfused and ventilated, areas marked by “••” were non-homogenously perfused, and “•••” areas were atelectatic. Results were summarized in F (mean +/- SD, p = 0,102; n = 5/group). Blood oxygenation measured 90min after LTX was significantly better in XIB13-treated animals compared to controls (G; mean +/- SD, p = 0,000); the correlation between preoperative tracheal edema with O_2_ saturation 90min post LTX is shown in (H, R² = 0,617, p = 0,004). To quantify vascular perfusion, biotinylated tomato-lectin was injected i.v. 5min before animals were sacrificed. The percentage of perfused vessels (marked by the lectin, green) was quantified in lung sections stained for desmin to identify large vessels (red), as exemplified in (I); scale bar 200μm. Data were summarized in (J, mean +/-SD, p = 0,287; n = 5/group); the vascular leak was quantified by extravasation of sodium fluorescein tracer injected simultaneously with lectin (K, mean +/- SD, p = 0,510; n = 5/group).

When the tracheal clamps were removed and the donor lungs deflated prior to anastomosis, tracheas expelled fluid, which was scored (absent, 0 to severe, 3). Representative images for score 0 and 3 are shown in Fig 1A and 1B.

#### Vascular leak and perfusion

Macroscopic appearances of lungs 100min post LTX were photo-documented. Perfused and ventilated areas were marked according to their color (bright red for well and homogenously perfused, dark-red for atelectatic and mottled for non-homogenously perfused as exemplified in Fig 1D and 1E). Lung areas were quantified with ImageJ blinded to the conditions based on color appearance. Subsequently areas were measured and calculated as percent of the total lung area.

To measure vascular perfusion, 100μg biotinylated lycopersicon esculentum lectin (Vector Laboratories Inc., Burlingame, CA) were injected, to measure vascular leak, 100μg sodium fluorescein in PBS (Sigma-Aldrich, St. Louis, MO) were injected i.v. 95min post LTX. Five minutes later, lungs were flushed with PBS (Lonza, Verviers, Belgium) and neutral buffered formalin (SAV LP GmbH, Flintsbach, Germany) and then explanted. Tip and median transverse sections were used for assessment of leakage of the fluorescent tracer. Tissues were weighed and broken up by milling in 1ml PBS. Samples were centrifuged and the amount of sodium fluorescein in supernatants was measured in a fluorescence spectrophotometer (excitation 485nm, emission 535nm, Tristar LB941, Berthold Technologies, Bad Wildbad, Germany). The remaining lung tissue was snap frozen, cryosectioned and subjected to immunolabelling. To reduce autofluorescence, sections were pretreated with 0,5% Pontamine Sky Blue solution. Muscular vessels were visualized by rabbit-anti-desmin antibody (Abcam, Cambridge, UK) followed by a TRITC labelled anti-rabbit antibody (Jackson ImmunoResearch, Suffolk, UK). The biotinylated lectin was visualized by incubation with Alexa 488 labelled streptavidin (Life Technologies, Eugene, OR) to visualize perfused vessels. Double and single positive vessels were counted by 2 independent observers blinded to the conditions.

#### Quantification of circulating donor DNA

DNA was isolated from peripheral blood. The gene ATP-binding cassette, subfamily B (MDR/TAP), member 10 has several nucleotide substitutions that are different between Wistar and Fisher rats. DNA was amplified using primers RatBShortFwd (5’-TTATGTATGTGAGTGCACTGTCG-3’) and RatBShortRev(5’-CATATTCAAACCACGCATCCCAG-3’) in a PCR reaction with Go Taq Long PCR mastermix (Promega Corporation, Madison, WI). Amplification was checked by Agarose gel electrophoresis. PCR samples were subsequently incubated with ExoSap-IT (Affymetrix, St. Clara, CA) to remove dNTPs and primers. An aliquot of 100ng of the amplification product was end repaired and adapters were ligated (IonPlus Fragment Library Kit, Ion Xpress Barcode Adaptors, Life Technologies, Carlsbad, CA). After several further purification steps (Agencourt® AMPure® XP—PCR product cleaning, Beckman-Coulter, Brea, CA) and agarose gel electrophoresis (E-Gel Size Select, Invitrogen, Kiryat Shmona, Israel) the template was recovered and the concentration determined (Qubit). Templates were loaded onto ionspheres; subsequently emulsion PCR with Ion PGM Template OT2 Reagents (Life Technologies, Carlsbad, CA) was performed. Afterwards spheres loaded with the clonally amplified library were enriched and mixed with the sequencing reagents (Ion PGM Sequencing Reagents 400bp, Life Technologies, Carlsbad, CA). This final mix was loaded onto a 314 Chip (Life Technologies, Carlsbad, CA) and a sequencing run on the Ion PGM device was performed. Sequence motif “GCA GCA GGT TCC CAA”, specific for Wistar rats and “GCA GCA GGC TCC CAA” specific for Fisher rats were quantified.

### Measurements in the chronic model, day 28 post LTX

#### Histology

**Fig 2:**
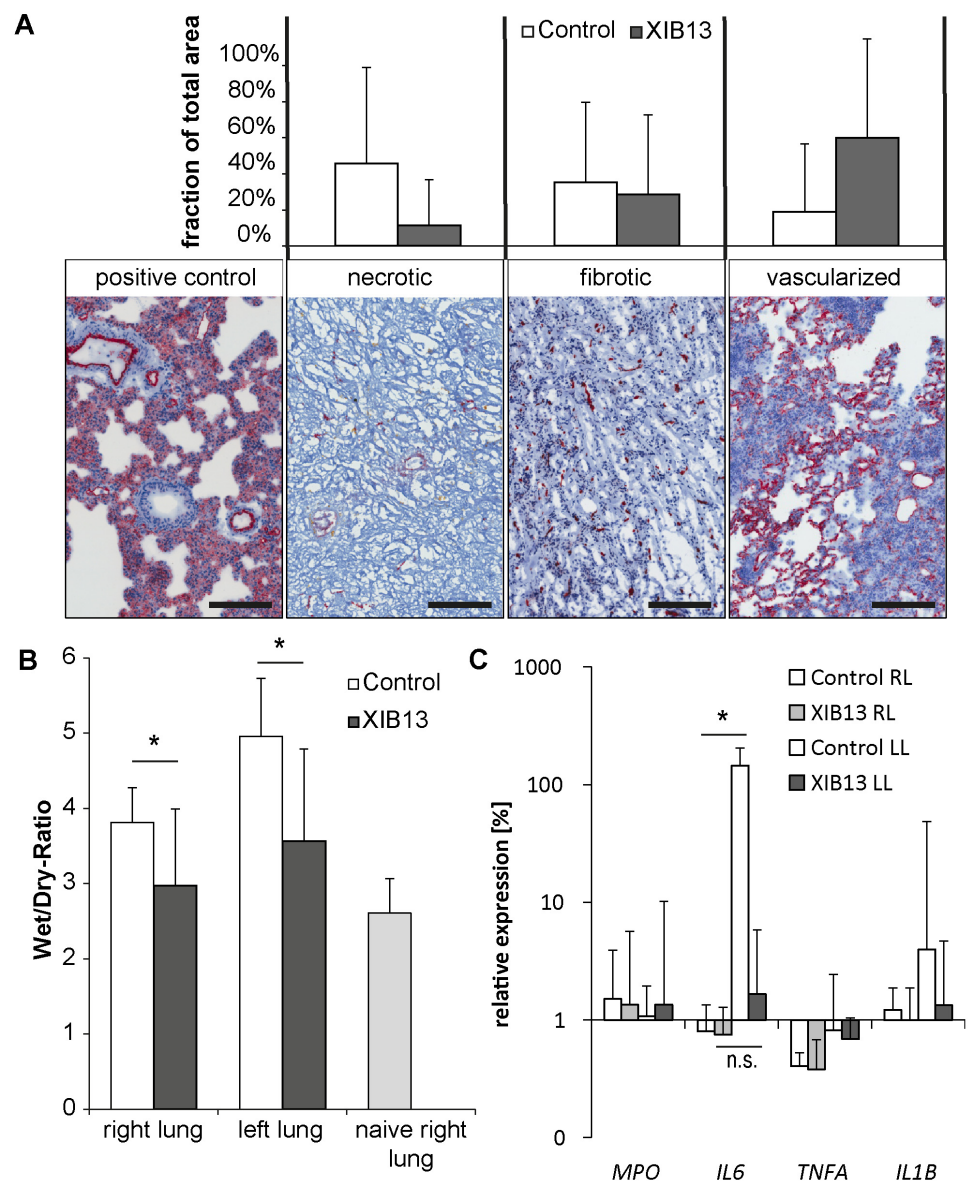
Improved tissue morphology and reduced edema in XIB13-treated lung grafts in rats 28 days post LTX. (A) 28 days post LTX, lung morphology in histological sections was graded as necrotic, fibrotic or with maintained alveolar structures in hematoxylin & eosin-stained sections combined with CD31 stains to identify vascularity of grafts (scale bar 200μm). Mean percent values and SD of necrotic, fibrotic or vascularized lung areas are shown for 5 animals per treatment group (p = 0,215); (B) To quantify tissue edema, wet-to-dry ratios of lungs were determined 28 days post LTX (mean +/- SD, right lungs p = 0,010, left lungs p = 0,003; n = 9/group); (C) mRNA expression of MPO, IL-6, TNFα and IL-1β in lung tissue of study animals. Values were expressed as fold change relative to untransplanted left lungs from donor animals (mean +/- SD; n = 5/group; * denotes p≤0,05; n.s. denotes not significant; p≥0,15 for all other targets).

Transverse lung sections from the tip and median region were used for cryo-sectioning. Sections were fixed in a 1:1 mixture of acetone and methanol. Tissue specimens were stained with hematoxylin and eosin or antibodies for CD31 (BD Biosciences). Slides were scanned with the Aperio system (Leica Biosystems, Nussloch, Germany) and areas with regular alveolar structures, or poorly vascularized and fibrotic areas or necrotic areas (as exemplified in Fig 2A) were marked in each lung section. The area covered by each classification group was measured and calculated as % of the total lung area.

#### Wet-to-dry-ratio

Wet-to-dry-ratio of the lungs was calculated from the weight immediately after excision and after drying to constant weight in a separate series of experiments (n = 9/group).

#### Quantitative RT-PCR

Lung tissue was lysed with RLT buffer and 1% β-mercaptoethanol by ball milling. Samples were further processed for isolation of RNA with Qiashredder (Qiagen, Venlo, Netherlands) and RNeasy Mini Kit (Qiagen) according to manufacturer’s instructions and reverse transcribed using RevertAid H Minus First Strand cDNA Synthesis Kit (Thermo Scientific, Waltham, USA). cDNA and Taqman PCR master mix were used with Taqman gene expression assays for respective targets (Life Technologies). The Ct threshold was set to 0.08 for all samples. Cts at threshold value were recorded and normalized to β-2-microglobulin. Data are shown as fold changes compared to untreated donor lungs.

#### Indirect Coombs test

Recipient serum from Wistar rats was incubated with isolated red blood cells from Fischer 344 rats and anti-rat antibody (Jackson ImmunoResearch). After 5 minutes at room temperature, cell agglutination was assessed micro- and macroscopically from triplicate wells of each sample. The results were the same for all triplicates and are reported as positive or negative agglutination reaction.

#### Mixed lymphocyte reaction (MLR)

Cells were isolated from rat spleens, red blood cells were removed by lysis. Donor spleen cells (antigen-presenting cells) were irradiated and 2x10^5^cells/well were mixed with indicated cell numbers from recipient spleens (effector cells) and were incubated at 37°C and 5% CO_2_. After 3 days 3-H thymidine was added for another 24h. Cells were harvested by Harvester96® (Tomtec, Hamdem, CT), scintillation liquid was added and 3-H thymidine incorporation was measured using Hidex-Chameleon (Hidex, Turku, Finland). Freshly isolated PBMCs were incubated with XIB13 or control peptide and proliferation index was assessed 3 days later by adding 3-H thymidine incorporation as previously described for spleen cells.

### Measurements in a ventilation mouse model

The Animal Care and Use Committee of the Academic Medical Center, Amsterdam, the Netherlands, approved the study. Animal procedures were performed in accordance with Institutional Standards for Human Care and Use of Laboratory Mice. Adult male C57Bl6 mice (N = 138; Charles River, Maastricht, the Netherlands), weighing 24–26g, received an intraperitoneal bolus of 1mL saline at 2h before the start of mechanical ventilation. A tracheotomy (Y–tube connector with 1,0mm outer diameter and 0,6mm inner diameter; VBM Medizintechnik GmbH, Sulz am Neckar, Germany) was inserted under general anesthesia with intraperitoneally 126mg/kg ketamine (Eurovet Animal Health BV, Bladel, the Netherlands), 0,2mg/kg medetomidine (Pfizer Animal Health BV, Capelle a/d IJssel, the Netherlands) and 0,5mg/kg atropine (Pharmachemie, Haarlem, the Netherlands). Mice were connected to a Servo Ventilator 900C (Siemens–Elema, Solna, Sweden) and ventilated for 4 hours in a pressure–controlled mode at a fractional inspired oxygen concentration (FiO_2_) of 0,5, inspiration–to–expiration ratio of 1:1 and positive end–expiratory pressure of 2cmH_2_O. Mechanical ventilation was applied with an inspiratory pressure of 10cmH_2_O (~ 7,5ml/kg). The respiratory rate was set at 100breaths/minute. Anaesthesia was maintained with 36mg/kg ketamine, 0,04mg/kg medetomidine, and 0,075mg/kg atropine, given hourly via an intraperitoneal catheter (PE 10 tubing; BD, Breda, the Netherlands). 200mmol/l NaHCO_3_ was administered every 30 minutes via the intraperitoneal catheter. Body temperature was kept steady between 36,5–37,5°C using a warming device. After 4 hours the right lung was lavaged by instilling 3 x 0,5 ml sterile saline into the trachea. Total cell counts were determined using a hemacytometer (Beckman Coulter, Fullerton, CA). The cell–free supernatant was used for ELISA. Wet/Dry-ratios were determined by weighing lungs immediately after excision and after drying to constant weight in a separate series of experiments (n = 9/group).

### Statistical Analysis

Data were analyzed with SPSS v. 21 (IBM Corp). The statistical evaluation was blinded to the conditions. Statistical significance for parametric data was assessed by Student’s t-test and statistical significance for nonparametric data was assessed by Wilcoxon-Mann-Whitney-Test; p≤0.05 was considered statistically significant. An ANOVA corrected for repeated measurements was used to evaluate significance of MLR data.

## Results

### The acute model (100min of reperfusion)

The first significant clinical finding was evident when the tracheal clamps were removed and the donor lungs deflated prior to anastomosis. Tracheas of random peptide-treated lungs expelled fluid, whereas this was not seen in most of the XIB13-treated tracheas, indicating less lung edema during the ischemia period. Examples are shown in Fig 1A and 1B; summarized scores of the whole cohort are displayed in 1C. After 100min of reperfusion, control lungs displayed large atelectatic and/or non-perfused areas, whereas most XIB13-treated lungs were fully reperfused and ventilated. Representative macroscopic images are shown in Fig 1D and 1E, and are summarized in 1F. Movies illustrating ventilation of grafted lungs 95min post LTX are exemplified in supplementary S1 and S2 Figs. S1 Fig shows a fully ventilated XIB13-treated lung, S2 Fig shows a poorly ventilated and partially atelectatic control lung. These findings were complemented by significantly improved blood oxygenation in the XIB13 group, which correlated with bronchus edema scores (Fig 1G and 1H). Lung perfusion of large muscular vessels, as determined by lectin injection and anti-desmin stains, was equal in both groups (Fig 1I and 1J). The extravasation of sodium fluorescein was lower in XIB13-treated rats, albeit not significant (Fig 1K). Lung inflammation was quantified in anti-myeloperoxidase-stained sections and revealed no differences between groups (data not shown).

### The chronic model (28 days of reperfusion)

Histological lung sections revealed that XIB13 treated animals had larger areas of maintained intact lung morphology as compared to controls, the latter having large necrotic or fibrotic zones (Fig 2A), but the amount of inflammation as determined semi-quantitatively, did not differ between groups (data not shown). The wet-dry-ratios of left lungs in XIB13-treated animals were significantly lower than in controls, almost comparable to naive lungs indicating reduced lung edema (Fig 2B). Unexpectedly, this was also seen in right lungs, which were not touched during the surgical procedure (Fig 2B) suggesting a persistent and systemic inflammatory response in controls. Therefore mRNA levels of inflammatory markers were assessed by quantitative RT-PCR (Fig 2C). The expression levels of IL-6 were significantly elevated in left compared to right lungs in control-treated animals, whereas IL-6 levels in XIB13-treated animals were low in both, right and left, lungs (difference not significant).

**Fig 3:**
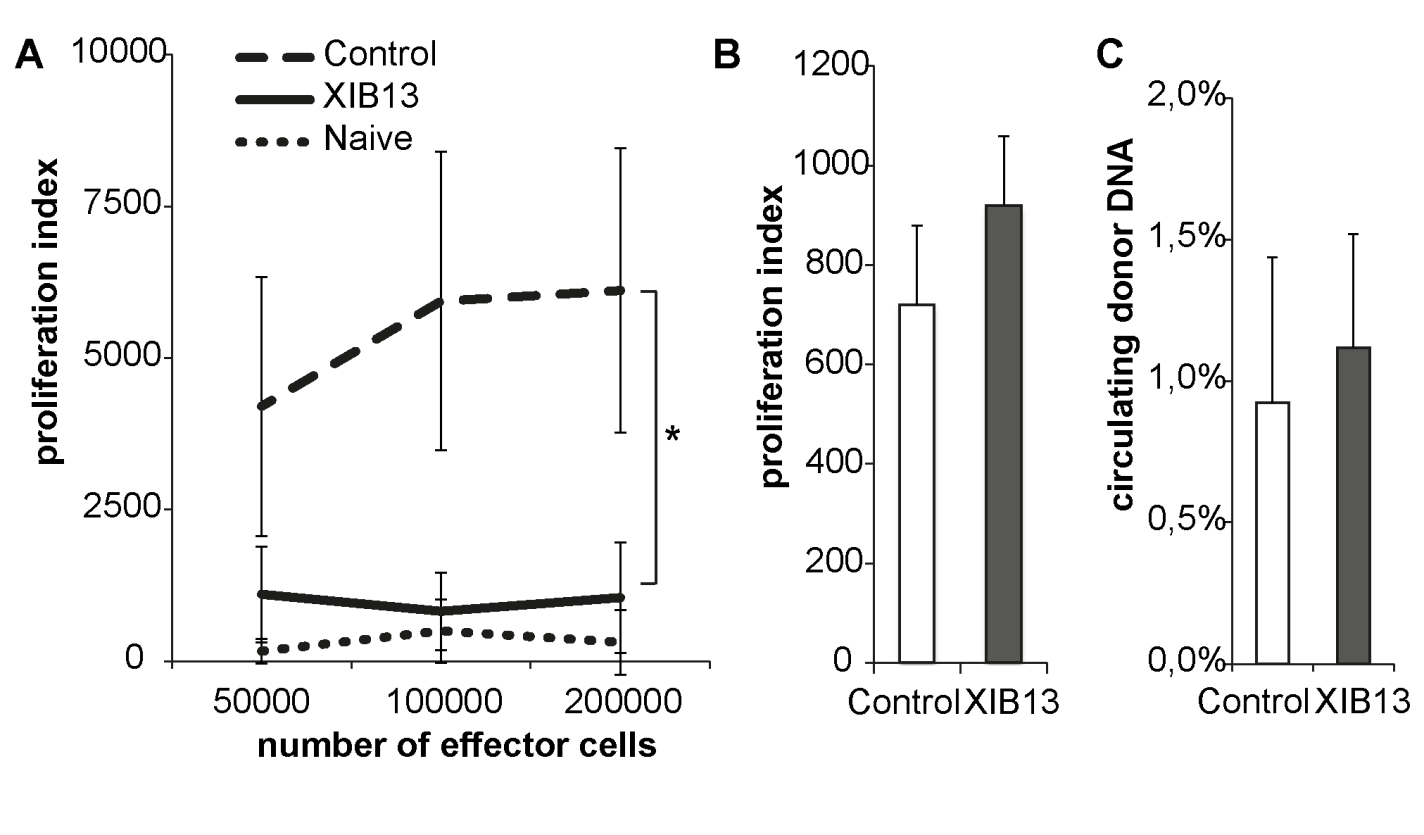
XIB13-treated rats have reduced allo-reactive T cells. T cells from recipient rats retrieved 28 days post LTX were mixed with irradiated spleen cells from donors and showed a significant reduction in proliferation as compared to control-treated animals (A, mean +/- SD, p = 0,000; n = 6). (B) The peptide did not directly alter lymphocyte proliferation: PBMCs were incubated with XIB13 or control peptide for 3 days (mean +/- SD, p = 0,178; n = 3); (C) The effect was not due to increased exposure of recipients to donor cells as measured by numbers of donor DNA copies in the recipients blood (B, mean +/- SD, p = 0,360; n = 6). XIB13 had no effect on Wet/Dry-ratios, cell influx and cytokine secretion in ventilation-induced lung injury in mice not subjected to any surgical procedure (Fig 4).

As a possibility, this could be attributed to the minor MHC mismatch between F344 and WKY rats, provoking an allogeneic response. An immunoglobulin-mediated rejection response was excluded by an indirect Coombs test (Table 1). However, testing for allo-reactive T cells, a profound proliferative response was obvious in spleen cells from controls and absent in cells derived from XIB13-treated rats (Fig 3A). This could not be attributed to an increased dissemination of donor cells from donor lungs into the recipients’ circulation as measured by the amount of circulating donor DNA (Fig 3C). Moreover, the peptide had no direct effect on T-cell proliferation, when added to PBMCs in vitro (Fig 3B).

**Fig 4:**
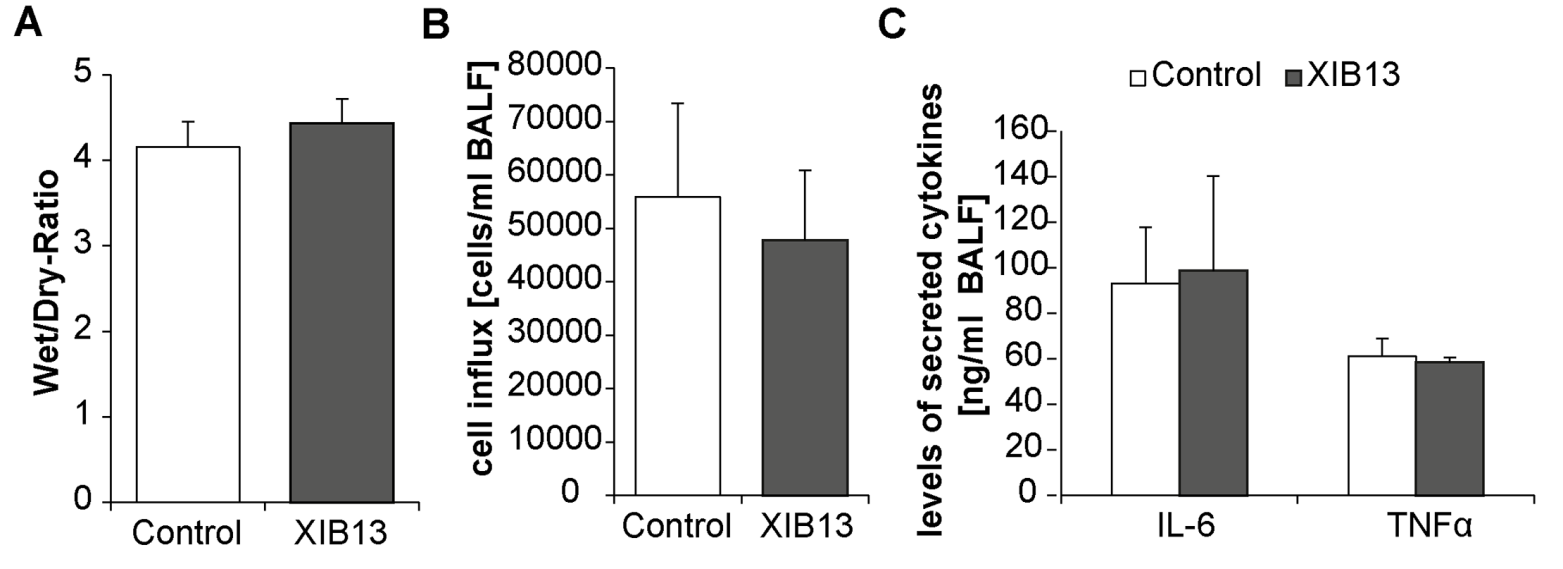
XIB13 has no effect on ventilation-induced lung injury in mice. (A) Tissue edema was quantified by determining Wet/Dry ratios of lungs (mean +/- SD, p = 0,059; n = 9/group); (B) cell influx was assessed in cytospin preparations of BALF (mean +/- SD, p = 0,337; n = 9/group); (C) cytokine levels (IL-6 and TNFα) in BALF were determined by ELISA. (mean +/- SD, p = 0,727 for IL-6, p = 0,190 for TNFα; n = 9/group).

**Table 1:** Positive agglutination by indirect Coombs test to assess anti-donor antibodies from recipient serum.

	100min		28days		syngeneic serum	human serum	PBS
	control	XIB13	control	XIB13			
positive	0/4	0/4	0/13	0/15	0/2	6/6	0/6

## Discussion

Lung transplantation (LTX) has become increasingly common, but survival rates consistently lag behind other organ transplantations. Specifically, primary graft dysfunction (PGD), a severe form of acute lung injury induced by ischemia-reperfusion injury, occurs in up to 50% of LTX recipients [18]. Reported 30-day mortality rates of patients with severe PGD are nearly 8 times as high as those for patients without PGD [19].

In our model of lung transplantation, the first clinically obvious finding was reduced expulsion of pulmonary fluid after the release of the tracheal clamp in donor lungs ex vivo-treated with a cingulin-derived peptide. XIB13-treated recipient animals then showed larger perfused and ventilated lung areas and improved oxygen saturation 100min post LTX, which correlated with reduced tracheal fluid expulsion. This indicates increased barrier function of alveolar capillaries and/or alveolar epithelium and was supported by reduced leakage of a small molecular tracer from the blood into the tissue. Due to the high standard deviation, which reflects the complexity of the procedure, it did not reach statistical significance. However, in our previous study in burns [16], where a highly reproducible tissue injury was introduced on mouse ears, we saw a significant and early reduction in burn-induced edema by XIB13-treatment administered as a continuous infusion in contrast to bolus injection in this study.

Concerning the 28 days follow up in LTX rats, we found improved maintenance of intact lung morphology and surprisingly, reduced lung edema. This could not be explained by the direct effects of XIB13, as this peptide has a half-life of ~5 minutes in human plasma and was only infused peri-operatively. As one possibility, we suggest reduced sensitization with allogeneic donor cells. In fact, 28 days post LTX we saw allogeneic T cell responses in control rats, but not in XIB13-treated rats. To test, if this correlated with increased circulating donor cells, we quantified chimerism, but found no differences. Interestingly, IL-6 mRNA expression in left lungs remained high after 28 days, indicating persistent activation, whereas IL-6 was low in both, left and right lungs in XIB13-treated animals. We speculate that the improved outcome in XIB13-treated animals is, in part, based on improved endothelial barrier function resulting in a reduced cellular exchange during or immediately after the transplantation procedure and in reduced sensitization.

The XIB-13 peptide closely resembles a sequence from the tight junction protein cingulin [16]. Cingulin is known to be expressed in epithelium, but to a lesser extent, is also found in endothelium (manuscript submitted). Cingulin keeps guanine nucleotide exchange factors (GEFs) in an inactive state, unable to activate RhoA [13]. Experiments to directly show that XIB13 interferes with scaffold-specific Rho activation requires FRET assays with RhoA biosensors, which allow time and site-specific visualization of RhoA activation at tight junctions (which we have not performed yet). However, the importance of Rho-GTPases as mediators of lung injury is well known. For example, the deletion of GEF-H1 or afadin genes reduces lung injury [20, 21]. Other approaches are aimed to stabilize junctional cadherin [22, 23], to enforce cellular adhesion [24, 25], to protect from oxidative stress [26] or to employ anti-inflammatory mediators [27, 28]. In one or the other way all these efforts aim to reduce endothelial activation in order to maintain barrier function [29, 30].

In conclusion, we provide evidence that the synthetic peptide XIB13 improves lung performance following LTX by increasing barrier function. This not only results in immediate increases in blood oxygenation and decreased edema, but during follow up also reduces sensitization of the host to donor MHC.

## Supporting Information

S1 Fig Video of improved inflation and perfusion in a XIB13-treated transplanted lung 95min post LTX. (MOV)

S2 Fig Video of inflation and perfusion in a random peptide -treated transplanted lung 95min post LTX. (MOV)

## Abbreviations

F344…: Fisher F344 donor rats
FiO2…: fraction of inspired oxygen
LTX…: lung transplantation
MLR…: mixed lymphocyte reaction
PEEP…: positive end-expiratory pressure
PGD…: primary graft dysfunction
WKY…: Wistar Kyoto recipient rats
